# Supplementary material for: Quantitative dose-response analysis untangles host bottlenecks to enteric infection
Source: Nat Commun. 2023 Jan 28;14:456. doi: 10.1038/s41467-023-36162-3 (PMC9884216; doi:10.1038/s41467-023-36162-3)
Supplement: Supplementary file 1 — Supplementary Information [file 41467_2023_36162_MOESM1_ESM.pdf]

Supplementary Information

Quantitative dose-response analysis untangles host bottlenecks to enteric infection

Ian W. Campbell, Karthik Hullahalli, Jerrold R. Turner, and Matthew K. Waldor*

*mwaldor@research.bwh.harvard.edu

Supplement 1

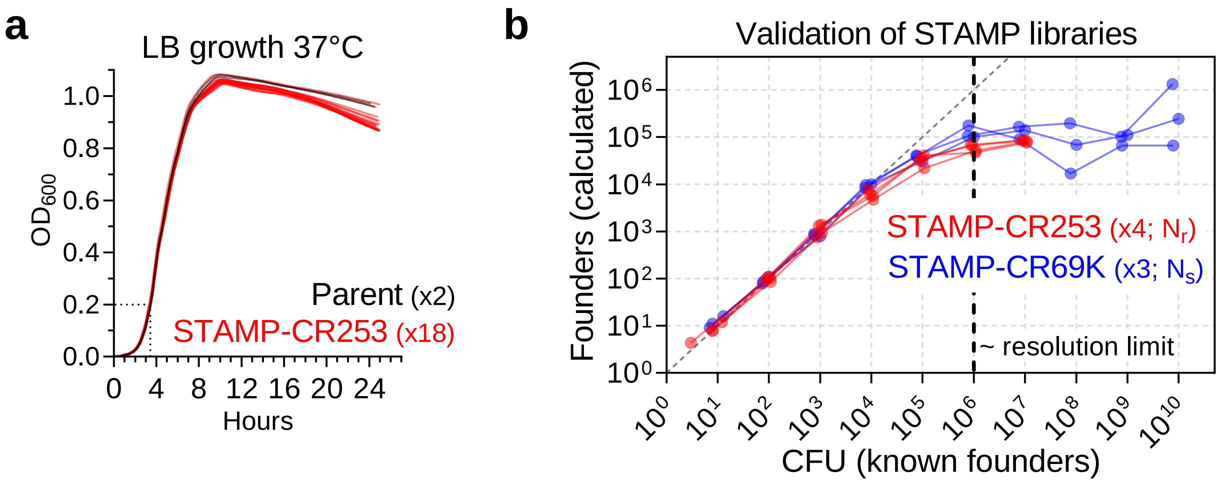

Supplemental figure 1. Validation of *Citrobacter rodentium* STAMP libraries.
(**a**) Growth of STAMP-CR253 library compared to the parental, untagged strain. Growth curve performed in 96 well plate reader at 37°C, shaking, in LB. 18 clones (colonies) from STAMP library and 2 from parent. Lines are means of triplicate samples.
(**b**) The ability to determine the size of the founding population from STAMP libraries was validated in culture by comparing the number of plated colonies (CFU; known number of founders) to the size of the founding population following processing of those plated populations (calculated founders). Without calibration, STAMP accurately determined the number of founders up to $\sim 10^4$ CFU and plateaued at $\sim 10^6$ CFU. Therefore, these values can be used to calibrate future experiments to determine the size of founding populations up to 10^6, with increased uncertainty above 10^4. STAMP-CR253 (253 unique barcodes) founders (N_r) measured for 4 cultures across 7 doses. STAMP-CR69K (~69,000 unique barcodes) founders (N_s) measured for 3 cultures across 10 doses.
Source data are provided as a Source Data file.

Supplement 2

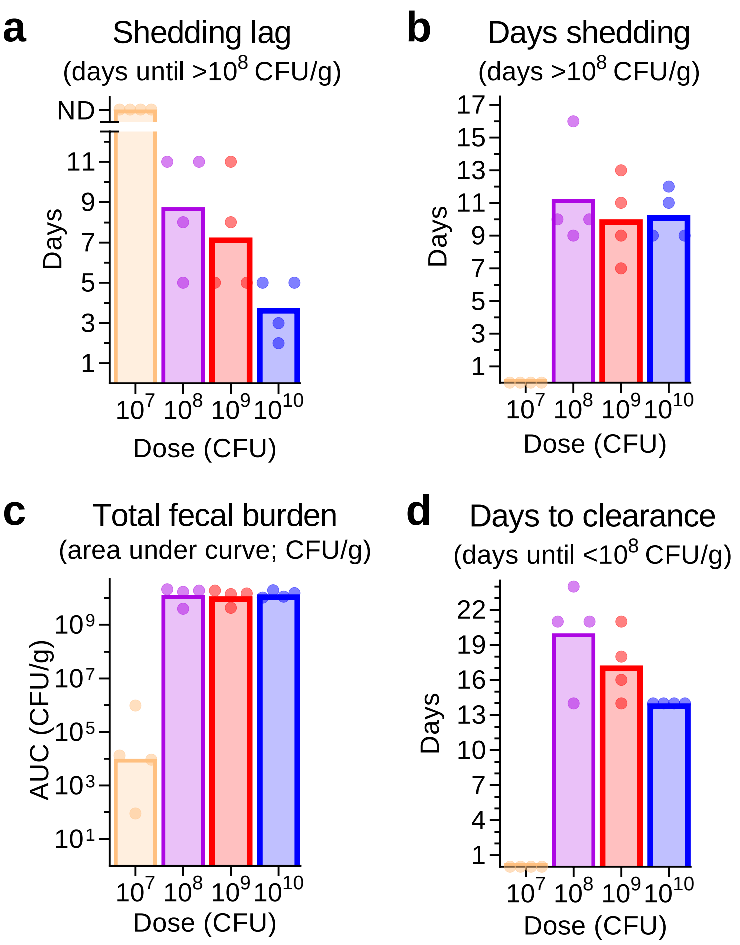

Supplemental figure 2. Additional data related to shedding of C57BL/6J mice in Figure 2.
In C57BL/6J mice inoculated with a high enough dose to cause infection (≥10^8 CFU), dose impacts the lag between inoculation and peak shedding (**a**) and the lag between inoculation and clearance (**d**) but does not change the days of peak shedding (**b**) or the total fecal burden (**c**). 4 animals per dose. (**a, b ,d**) Arithmetic mean, (**c**) geometric mean.
Source data are provided as a Source Data file.

Supplement 3

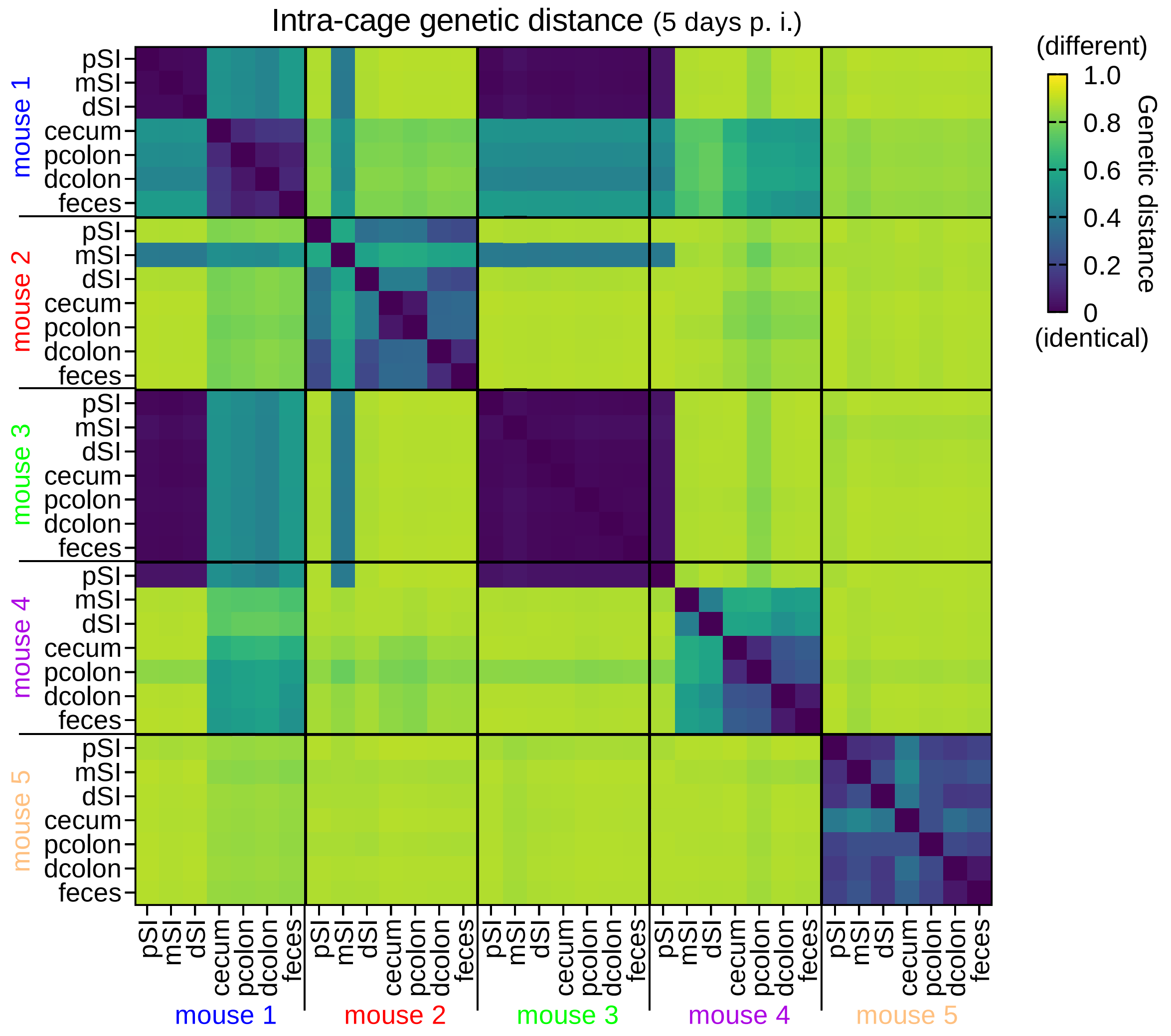

Supplemental figure 3. All intra-cage relationships from Figure 4a-e.
Comparison by genetic distance of *C. rodentium* populations 5 days post inoculation in 5 cohoused (intra-cage) C57BL/6J mice inoculated with STAMP-CR253. Zero indicates no difference between populations (identical). From proximal (p) to distal (d): p small intestine (SI), mid (m) SI, dSI, cecum, pcolon, dcolon, feces. Source data are provided as a Source Data file.

Supplement 4

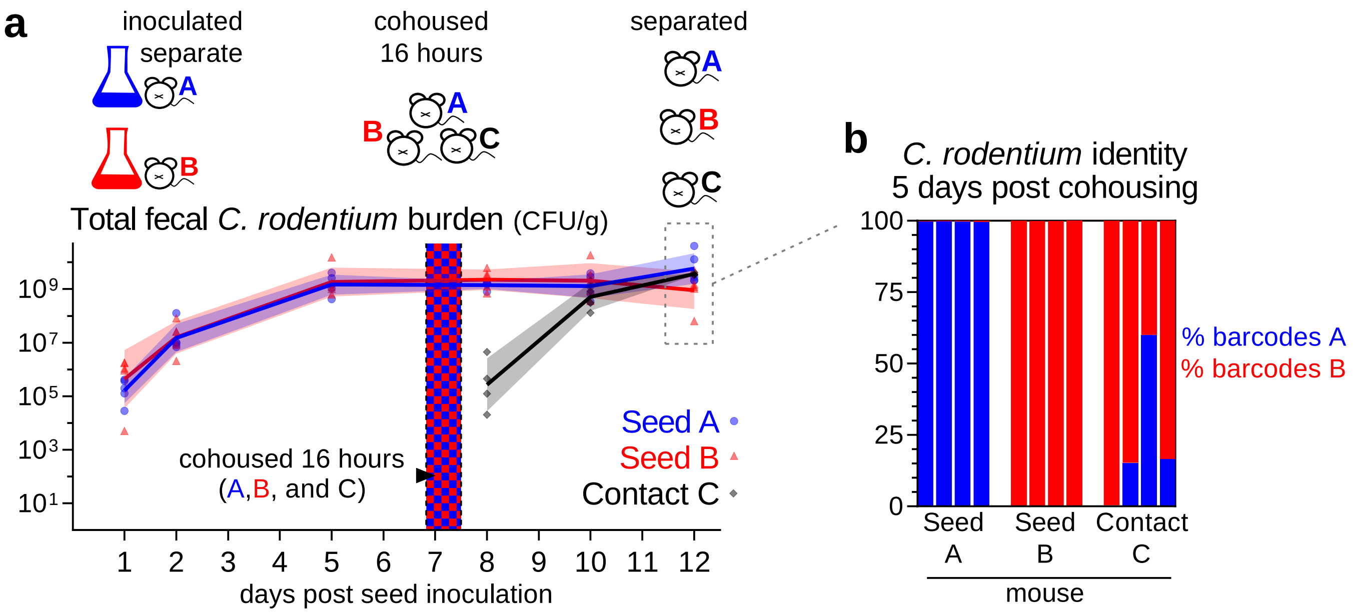

Supplemental figure 4. Super-colonization resistance prevents cohoused mice from sharing *C. rodentium* populations.

To test transmission of *C. rodentium* to already infected mice, two cages of 4 C57BL/6J mice (seeds A and B) were separately inoculated with mixed pools of barcoded strains (barcodes A and B). Barcodes A and B are each a group of 3 separate strains with non-redundant barcodes. Seed animals A and B were separated until peak colonization. On the 6th day after seed inoculation, the mice were re-arranged to cohouse 1 mouse each of seed A, seed B, and uninfected contact C overnight for 16 hours. The next morning, they were separated, housing the A seeds together, the B seeds together, and the C contacts together. 12 days post seed inoculation (5 days post cohousing) the fecal *C. rodentium* populations were analyzed to determine the contribution from pools A and B. <1% of reads from seed A mice were contributed by the B barcodes and vice versa. (**a**) Shedding data is presented as geometric means and standard deviations. Source data are provided as a Source Data file.

Supplement 5

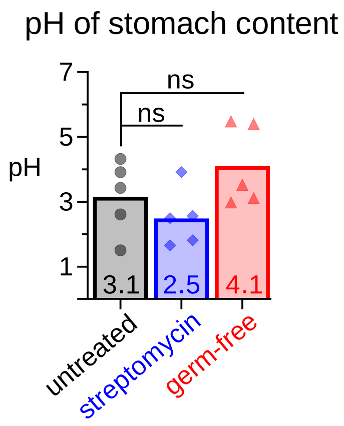

Supplemental figure 5. The effect of microbiota on gastric acid.
Conventional, untreated C57BL/6J mice have similar stomach acidity as mice treated for 3 days with the antibiotic streptomycin (as in Figure 6) or mice lacking a microbiota (germ-free, as Figure 7). pH measured post-mortem in aspirated stomach fluid. Boxes are arithmetic means. One-way ANOVA adjusted *p*-values 0.54 (streptomycin) and 0.32 (germ-free), not significant (ns). 5 animals per group.
Source data are provided as a Source Data file.

Supplemental table 1

microbiota	days p. i.	cage	mouse	variation	genomic change	annotation	coding change	amino acid change
SPF C57BL/6J	5 days	A	A1	complex	g.3081146..3081467	intergenic		
			A2	none				
			A3	SNV	g.388509C>G	*lamB*	c.766G>C	p.Val256Leu
			A4	none				
		B	B1	none				
			B2	none				
germ-free C57BL/6J		C	C1	SNV	g.527543C>T	*panE*	c.232G>A	p.Asp78Asn
			C2	none				
			C3	SNV	g.330872T>C	*ROD_03001*	c.557A>G	p.Glu186Gly
				SNV	g.4274290A>G	*dgoT*	c.442A>G	p.Arg148Gly
			C4	none				
	20 days		C1	deletion	g.3100349..3180553	multiple (including LEE)		
				SNV	g.4029101C>T	*glpK*	c.1000C>T	p.Pro334Ser
				SNV	g.5295383C>T	intergenic		
			C2	deletion	g.3100349..3180553	multiple (including LEE)		
				SNV	g.4029101C>T	*glpK*	c.1000C>T	p.Pro334Ser
				SNV	g.5295383C>T	intergenic		
			C3	deletion	g.3100349..3180553	multiple (including LEE)		
				SNV	g.4029101C>T	*glpK*	c.1000C>T	p.Pro334Ser
				SNV	g.5295383C>T	intergenic		
			C4	deletion	g.3100349..3180553	multiple (including LEE)		
				SNV	g.4029101C>T	*glpK*	c.1000C>T	p.Pro334Ser
				SNV	g.5295383C>T	intergenic		
		D	D1	SNV	g.4270455T>A	*dgoR*	c.143T>A	p.Val48Glu
				SNV	g.4274290A>G	*dgoT*	c.442A>G	p.Arg148Gly
				SNV	g.4634927C>T	*gntU*	c.895C>T	non-coding
				insertion	g.5407322^5407357	intergenic		
		E	E1	deletion	g.3100354…3197823	multiple (including LEE)		
				insertion	g.4989831^4989834	*gatZ*		
		F	F1	deletion	g.3100357..3198048	multiple (including LEE)		
				SNV	g.3937003C>T	*purH*	c.837C>T	non-coding
				SNV	g.4274290A>G	*dgoT*	c.442A>G	p.Arg148Gly
		G	G1	deletion	g.2391346..2391356	*galS*		
				complex	g.3174563..3174662	*grlR*		

Supplemental table 1. Changes in the *C. rodentium* genome following mouse expansion. Whole genome sequencing was used to detect variations in the *C. rodentium* genome after expansion and selection in mice. 3 clones (colonies) from the STAMP-CR69K library were compared to isolates from the feces of infected mice (1 colony per mouse). No variants ("none") detected in 4/6 isolates from SPF (specific pathogen free) animals 5 days p. i., 2/4 isolates from germ-free animals 5 days p. i., and 0/8 isolates from germ-free animals 20 days p.i. 4 cohoused germ-free animals 20 days p. i. (C1-4) had identical (clonal) isolates. These data were filtered to only include high confidence variants (present in >90% of reads).

Supplemental table 2

Aim	5' -> 3'
Clone ~800bp of *C. rodentium* intergenic DNA into pKD4	cttcggaataggaactaaggaggatattcaGTCTGACGCCGGTATCTGTAATGC
	acggctgacatgggaattagccatggtccaAAAAAGCGCCGGGTGGCG
Check insertion into pKD4	gcgggactctggggttcgaa
	cgcagttcaacctgttgata
Clone *C. rodentium* ~800bp and KanR from pKD4 into pDS132 with 20n	ATATGTGATGGGTTAAAAAGGATCGATCCTnnnnnnnnnnnnnnnnnnnntcacgctgccgcaagcactc
	cgggagagctcgatatcgcatgcggtacctaaaaagcgccgggtggcggcttcgc
Check insertion into pDS132	CAGAAAATATCATAATATCT
	ACATGTGGAATTGTGAGCGG
	GATTTGCAGACTACGGGCCTAAAG
	GTGAGCGGATAACAATTTGTGG
Check integration into genome	GATTTGCAGACTACGGGCCT
	ctggaggatgaggggcggatgattggctcgaaccatctgc

Supplemental table 2. Primers used in the creation of STAMP-CR253.

Supplemental table 3

Name	5' -> 3'
AD001	CAAGCAGAAGACGGCATACGAGATCGTGATGTGACTGGAGTTCAGACGTGTGCTCTTCCGATCAGATCCTTGGCGGCAAGAAA
AD002	CAAGCAGAAGACGGCATACGAGATACATCGGTGACTGGAGTTCAGACGTGTGCTCTTCCGATCAGATCCTTGGCGGCAAGAAA
AD003	CAAGCAGAAGACGGCATACGAGATGCCTAAGTGACTGGAGTTCAGACGTGTGCTCTTCCGATCAGATCCTTGGCGGCAAGAAA
AD004	CAAGCAGAAGACGGCATACGAGATTGGTCAGTGACTGGAGTTCAGACGTGTGCTCTTCCGATCAGATCCTTGGCGGCAAGAAA
AD005	CAAGCAGAAGACGGCATACGAGATCACTGTGTGACTGGAGTTCAGACGTGTGCTCTTCCGATCAGATCCTTGGCGGCAAGAAA
AD006	CAAGCAGAAGACGGCATACGAGATATTGGCGTGACTGGAGTTCAGACGTGTGCTCTTCCGATCAGATCCTTGGCGGCAAGAAA
AD007	CAAGCAGAAGACGGCATACGAGATGATCTGGTGACTGGAGTTCAGACGTGTGCTCTTCCGATCAGATCCTTGGCGGCAAGAAA
AD008	CAAGCAGAAGACGGCATACGAGATTCAAGTGTGACTGGAGTTCAGACGTGTGCTCTTCCGATCAGATCCTTGGCGGCAAGAAA
AD009	CAAGCAGAAGACGGCATACGAGATCTGATCGTGACTGGAGTTCAGACGTGTGCTCTTCCGATCAGATCCTTGGCGGCAAGAAA
AD010	CAAGCAGAAGACGGCATACGAGATAAGCTAGTGACTGGAGTTCAGACGTGTGCTCTTCCGATCAGATCCTTGGCGGCAAGAAA
AD011	CAAGCAGAAGACGGCATACGAGATGTAGCCGTGACTGGAGTTCAGACGTGTGCTCTTCCGATCAGATCCTTGGCGGCAAGAAA
AD012	CAAGCAGAAGACGGCATACGAGATTACAAGGTGACTGGAGTTCAGACGTGTGCTCTTCCGATCAGATCCTTGGCGGCAAGAAA
AD013	CAAGCAGAAGACGGCATACGAGATTTGACTGTGACTGGAGTTCAGACGTGTGCTCTTCCGATCAGATCCTTGGCGGCAAGAAA
AD014	CAAGCAGAAGACGGCATACGAGATGGAACTGTGACTGGAGTTCAGACGTGTGCTCTTCCGATCAGATCCTTGGCGGCAAGAAA
AD015	CAAGCAGAAGACGGCATACGAGATTGACATGTGACTGGAGTTCAGACGTGTGCTCTTCCGATCAGATCCTTGGCGGCAAGAAA
AD016	CAAGCAGAAGACGGCATACGAGATGGACGGGTGACTGGAGTTCAGACGTGTGCTCTTCCGATCAGATCCTTGGCGGCAAGAAA
AD018	CAAGCAGAAGACGGCATACGAGATGCGGACGTGACTGGAGTTCAGACGTGTGCTCTTCCGATCAGATCCTTGGCGGCAAGAAA
AD019	CAAGCAGAAGACGGCATACGAGATTTTCACGTGACTGGAGTTCAGACGTGTGCTCTTCCGATCAGATCCTTGGCGGCAAGAAA
AD020	CAAGCAGAAGACGGCATACGAGATGGCCACGTGACTGGAGTTCAGACGTGTGCTCTTCCGATCAGATCCTTGGCGGCAAGAAA
Var11	AATGATACGGCGACCACCGAGATCTACACTCTTTCCCTACACGACGCTCTTCCGATCTGATGGGTTAAAAAGGATCGATCC
Var12	AATGATACGGCGACCACCGAGATCTACACTCTTTCCCTACACGACGCTCTTCCGATCTAGATGGGTTAAAAAGGATCGATCC
Var13	AATGATACGGCGACCACCGAGATCTACACTCTTTCCCTACACGACGCTCTTCCGATCTTACGATGGGTTAAAAAGGATCGATCC
Var14	AATGATACGGCGACCACCGAGATCTACACTCTTTCCCTACACGACGCTCTTCCGATCTGCTAGATGGGTTAAAAAGGATCGATCC
Var15	AATGATACGGCGACCACCGAGATCTACACTCTTTCCCTACACGACGCTCTTCCGATCTCGACTGGATGGGTTAAAAAGGATCGATCC
Var16	AATGATACGGCGACCACCGAGATCTACACTCTTTCCCTACACGACGCTCTTCCGATCTTAGCGTAGATGGGTTAAAAAGGATCGATCC
Var17	AATGATACGGCGACCACCGAGATCTACACTCTTTCCCTACACGACGCTCTTCCGATCTTGCTGAGTCGATGGGTTAAAAAGGATCGATCC
Var18	AATGATACGGCGACCACCGAGATCTACACTCTTTCCCTACACGACGCTCTTCCGATCTGCTGATAGTAGATGGGTTAAAAAGGATCGATCC
Var19	AATGATACGGCGACCACCGAGATCTACACTCTTTCCCTACACGACGCTCTTCCGATCTCAACTGCGTAGCGATGGGTTAAAAAGGATCGATCC

Var20	AATGATACGGCGACCACCGAGATCTACACTCTTTCCCTACACGACGCTCTTCCGATCTATGACCGTCT CGAGATGGGTTAAAAAGGATCGATCC
var21	AATGATACGGCGACCACCGAGATCTACACTCTTTCCCTACACGACGCTCTTCCGATCTAATGATGGGT TAAAAAGGATCGATCC
var22	AATGATACGGCGACCACCGAGATCTACACTCTTTCCCTACACGACGCTCTTCCGATCTATGCGATGGG TTAAAAAGGATCGATCC
var23	AATGATACGGCGACCACCGAGATCTACACTCTTTCCCTACACGACGCTCTTCCGATCTTGCACGATGG GTTAAAAAGGATCGATCC
var24	AATGATACGGCGACCACCGAGATCTACACTCTTTCCCTACACGACGCTCTTCCGATCTTCATTCGATG GGTTAAAAAGGATCGATCC
var25	AATGATACGGCGACCACCGAGATCTACACTCTTTCCCTACACGACGCTCTTCCGATCTGAATCGAGAT GGGTTAAAAAGGATCGATCC
var26	AATGATACGGCGACCACCGAGATCTACACTCTTTCCCTACACGACGCTCTTCCGATCTGTCAACTTGAT GGGTTAAAAAGGATCGATCC
var27	AATGATACGGCGACCACCGAGATCTACACTCTTTCCCTACACGACGCTCTTCCGATCTCGGCGTGGCG ATGGGTTAAAAAGGATCGATCC
var28	AATGATACGGCGACCACCGAGATCTACACTCTTTCCCTACACGACGCTCTTCCGATCTCCTGTACCTT GATGGGTTAAAAAGGATCGATCC

Supplemental table 3. Primers used for STAMP sequencing.
