# Supplementary material for: Quantitative dose-response analysis untangles host bottlenecks to enteric infection
Source: Nat Commun. 2023 Jan 28;14:456. doi: 10.1038/s41467-023-36162-3 (PMC9884216; doi:10.1038/s41467-023-36162-3)
Supplement: Supplementary file 3 — Reporting Summary [file 41467_2023_36162_MOESM3_ESM.pdf]

nature portfolio

Corresponding author(s): Ian W. Campbell, Matthew K. Waldor

Last updated by author(s): 01/09/2022

Reporting Summary

Nature Portfolio wishes to improve the reproducibility of the work that we publish. This form provides structure for consistency and transparency in reporting. For further information on Nature Portfolio policies, see our Editorial Policies and the Editorial Policy Checklist.

Statistics

For all statistical analyses, confirm that the following items are present in the figure legend, table legend, main text, or Methods section.

n/a	Confirmed	
☐	☒	The exact sample size (*n*) for each experimental group/condition, given as a discrete number and unit of measurement
☐	☒	A statement on whether measurements were taken from distinct samples or whether the same sample was measured repeatedly
☐	☒	The statistical test(s) used AND whether they are one- or two-sided<br>*Only common tests should be described solely by name; describe more complex techniques in the Methods section.*
☒	☐	A description of all covariates tested
☐	☒	A description of any assumptions or corrections, such as tests of normality and adjustment for multiple comparisons
☐	☒	A full description of the statistical parameters including central tendency (e.g. means) or other basic estimates (e.g. regression coefficient) AND variation (e.g. standard deviation) or associated estimates of uncertainty (e.g. confidence intervals)
☐	☒	For null hypothesis testing, the test statistic (e.g. *F*, *t*, *r*) with confidence intervals, effect sizes, degrees of freedom and *P* value noted<br>*Give P values as exact values whenever suitable.*
☒	☐	For Bayesian analysis, information on the choice of priors and Markov chain Monte Carlo settings
☒	☐	For hierarchical and complex designs, identification of the appropriate level for tests and full reporting of outcomes
☒	☐	Estimates of effect sizes (e.g. Cohen's *d*, Pearson's *r*), indicating how they were calculated

Our web collection on statistics for biologists contains articles on many of the points above.

Software and code

Policy information about availability of computer code

Data collection	For STAMP sequencing, data was collected using an Illumina MiSeq and FASTQ files were generated by Illumina's proprietary analysis pipeline (FASTQ Generation V1.0.0). For bacterial whole genome sequencing, data was collected by MiGS using an Illumina NextSeq and FASTQ files generated as above.
Data analysis	STAMP FASTQ files were processed with CLC Genomics Workbench (Qiagen) and Geneious (Biomatters) to create a barcode table with the number of reads mapping to each barcode in each sample. The barcode tables were then analyzed using custom scripts. Scripts and barcode tables are provided on github at hgttps://github.com/hullahalli/stampr_rtisan (doi 10.5281/zenodo.7521417).<br>Bacterial whole genome FASTQ files were mapped to the C. rodentium ICC168 reference genome NCBI ASM2708v1 [https://www.ncbi.nlm.nih.gov/assembly/GCA_000027085.1] with CLC Genomics Workbench (Qiagen). Reads from whole genome sequencing are available at the Sequencing Read Archive under PRJNA895384 [https://www.ncbi.nlm.nih.gov/bioproject/?term=PRJNA895384]. Variant detection was also performed with CLC.<br>Statistical analyses were performed using GraphPad Prism version 9.

For manuscripts utilizing custom algorithms or software that are central to the research but not yet described in published literature, software must be made available to editors and reviewers. We strongly encourage code deposition in a community repository (e.g. GitHub). See the Nature Portfolio guidelines for submitting code & software for further information.

Data

Policy information about availability of data

All manuscripts must include a data availability statement. This statement should provide the following information, where applicable:

- Accession codes, unique identifiers, or web links for publicly available datasets
- A description of any restrictions on data availability
- For clinical datasets or third party data, please ensure that the statement adheres to our policy

STAMP scripts and barcode read counts from this study are deposited online at https://github.com/hullahalli/stampr_rtisan. Reads from bacterial whole genome sequencing are available in the Sequencing Read Archive (BioProject accession PRJNA895384).

Human research participants

Policy information about studies involving human research participants and Sex and Gender in Research.

Reporting on sex and gender	N/A
Population characteristics	N/A
Recruitment	N/A
Ethics oversight	N/A

Note that full information on the approval of the study protocol must also be provided in the manuscript.

Field-specific reporting

Please select the one below that is the best fit for your research. If you are not sure, read the appropriate sections before making your selection.

☒ Life sciences ☐ Behavioural & social sciences ☐ Ecological, evolutionary & environmental sciences

For a reference copy of the document with all sections, see nature.com/documents/nr-reporting-summary-flat.pdf

Life sciences study design

All studies must disclose on these points even when the disclosure is negative.

Sample size	Sample sizes were determined by the variation observed during preliminary experiments.
Data exclusions	STAMP samples were excluded after sequencing if they received less than 100,000 reads unless it was apparent that less than 1,000 barcodes were present in the sample.
Replication	All individual points in display items are independent biological replicates (a sample or measurement collected from an independent animal or culture). Notably, we found similar results using two independent C. rodentium STAMP libraries. Figure 1B, Figure 2 B-C, and Figure 3 C-D (plus supplemental Figure 2 and 3) contain consistent data for infection dynamics (shedding and founding population) of untreated B6 mice challenged with two independent C. rodentium STAMP libraries. Figure 3A is 7 and 5 animals and the results are consistent with Supplemental Figure 5. Figure 3B is 10 independent cultures, which display a consistent pattern across multiple doses. Figure 3 C-D "Loxtidine" are 16 independent animals with a consistent trend across doses. Figure 4 A-E is a single cage of 5 animals, which produce consistent results with the 22 animals in Figure 4 F-G. Figure 5 C3H/HeOuJ are 12 animals with a consistent trend across 3 doses. Figure 6 is 20 animals with a consistent trend across 5 doses. Figure 7 is 20 animals with a consistent trend across 5 doses. Supplemental Figure 1A is 20 independent cultures in triplicate. Supplemental Figure 1B is 7 independent cultures. Supplemental Figure 4 is 4 independent co-housing of 3 mice each. The control of Supplemental Figure 5 is consistent with Figure 3A and streptomycin-treated and germ-free are each 5 independent animals. All attempts at replication were successful.
Randomization	Mice were randomly assigned to experimental groups.
Blinding	Blinding was not used in these studies as most values were measured by sequencing, which is independent of the investigator, and all measurements were at defined timepoints.

Reporting for specific materials, systems and methods

We require information from authors about some types of materials, experimental systems and methods used in many studies. Here, indicate whether each material, system or method listed is relevant to your study. If you are not sure if a list item applies to your research, read the appropriate section before selecting a response.

Materials & experimental systems

n/a	Involved in the study	
☒	☐	Antibodies
☒	☐	Eukaryotic cell lines
☒	☐	Palaeontology and archaeology
☐	☒	Animals and other organisms
☒	☐	Clinical data
☒	☐	Dual use research of concern

Methods

n/a	Involved in the study	
☒	☐	ChIP-seq
☒	☐	Flow cytometry
☒	☐	MRI-based neuroimaging

Animals and other research organisms

Policy information about studies involving animals; ARRIVE guidelines recommended for reporting animal research, and Sex and Gender in Research

Laboratory animals	Mice were C57BL/6J or C3H/HeOuJ and 9-14 weeks of age at the start of experimentation. Mice were housed in a temperature (68-75F) and humidity (50%) controlled facility with 12 hour light/dark cycles.
Wild animals	This study did not involve wild animals.
Reporting on sex	All mice were female.
Field-collected samples	This study did not include field samples.
Ethics oversight	All experiments involving mice were performed according to protocols reviewed and approved by the Brigham and Women's Hospital Institutional Animal Care and Use Committee (protocol 2016N000416) and in compliance with the Guide for the Care and Use of Laboratory Animals.

Note that full information on the approval of the study protocol must also be provided in the manuscript.
